# Role of latent membrane protein 1 in chronic active Epstein–Barr virus infection-derived T/NK-cell proliferation

**DOI:** 10.1002/cam4.256

**Published:** 2014-05-03

**Authors:** Takuto Ito, Hidetaka Kawazu, Takayuki Murata, Seiko Iwata, Saki Arakawa, Yoshitaka Sato, Kiyotaka Kuzushima, Fumi Goshima, Hiroshi Kimura

**Affiliations:** 1 Department of Virology, Nagoya University Graduate School of Medicine65 Tsurumai-cho, Showa-ku, Nagoya, 466-8550, Japan; 2 Division of Immunology, Aichi Cancer Center Research Institute1-1 Kanokoden, Chikusa-ku, Nagoya, 464-8681, Japan

**Keywords:** AKT, CAEBV, dominant negative, LMP1, NF*κ*B

## Abstract

Epstein–Barr virus (EBV) predominantly infects B cells and causes B-cell lymphomas, such as Burkitt lymphoma and Hodgkin lymphoma. However, it also infects other types of cells, including T and natural killer (NK) cells, and causes disorders, such as chronic active EBV infection (CAEBV) and T/NK-cell lymphoma. The CAEBV is a lymphoproliferative disease with poor prognosis, where EBV-positive T or NK cells grow rapidly, although the molecular mechanisms that cause the cell expansion still remain to be elucidated. EBV-encoded latent membrane protein 1 (LMP1) is an oncogene that can transform some cell types, such as B cells and mouse fibroblasts, and thus may stimulate cell proliferation in CAEBV. Here, we examined the effect of LMP1 on EBV-negative cells using the cells conditionally expressing LMP1, and on CAEBV-derived EBV-positive cells by inhibiting the function of LMP1 using a dominant negative form of LMP1. We demonstrated that LMP1 was responsible for the increased cell proliferation in the cell lines derived from CAEBV, while LMP1 did not give any proliferative advantage to the EBV-negative cell line.

## Introduction

Epstein–Barr virus (EBV) is a ubiquitous human virus that belongs to the *γ*-herpesvirus subfamily. A primary, acute infection of EBV in adolescence can cause infectious mononucleosis, and the virus is also associated with many types of tumor, including Burkitt lymphoma, nasopharyngeal carcinoma (NPC), and posttransplant lymphoproliferative disorder 1,2. EBV generally infects B cells via CD21 on the cell surface 3,4, but it can also rarely infect T or natural killer (NK) cells by unknown, CD21-independent mechanisms 5. The infection of T or NK cells can cause diseases with poor prognosis, including chronic active EBV infection (CAEBV), extranodal NK/T-cell lymphoma (ENKTL), nasal type and aggressive NK-cell leukemia (ANKL) 5.

CAEBV occasionally results in severe, chronic or recurrent infectious mononucleosis-like symptoms, such as fever, persistent hepatitis, extensive lymphadenopathy, hepatosplenomegaly, and pancytopenia, and has high mortality 6. Despite its severity, the therapeutic options are limited and adequate therapies are not yet established; patients are currently treated with anti-cancer or immunoregulatory drugs and/or a bone marrow transplant. Recent reports have suggested that bone marrow transplants give promising results, but this is a high-risk procedure and so the development of safe, effective, and specific alternative therapies remains important. The development of novel drugs for the treatment of CAEBV has been hampered by a limited number of cases, and also by a poor understanding of the disease pathogenesis. Therefore, understanding the molecular mechanisms of the dysregulated cell proliferation in CAEBV is critical. The clonal expansion of EBV-infected T or NK cells during the development and maintenance of CAEBV is the major factor that contributes to poor prognosis. We are thus determined to identify the factors responsible for the dysregulated cell division of T or NK cells in this lymphoproliferative disorder.

In cells transformed by EBV, the virus exists in a latent infection state which is characterized by a limited expression of viral proteins and RNAs 1. Neoplasms such as Burkitt lymphoma or gastric carcinoma typically express only the EBV-encoded RNAs (EBERs) and EBV nuclear antigen 1 (EBNA1) (latency type I), whereas some Hodgkin lymphoma, NPC, and T/NK lymphomas produce EBERs, EBNA1, and latent membrane protein 1 (LMP1) and LMP2 genes (latency type II). In addition to type II genes, EBNA2, EBNA3, and EBNA-LP are also expressed in most cases of immunosuppression-related lymphomas and lymphoblastoid cell lines (LCLs) (latency type III) 1,5.

One of the EBV-encoded genes expressed in the latency II or III state, LMP1 is an oncogene that plays a well-established role in B lymphocytic tumors and NPC 2,7,8. LMP1 can transform a several cell types by mimicking the activated form of human CD40 9,10. LMP1 is a membrane protein with tandem six transmembrane domains and C-terminal signaling domains (please see Fig. 4A). The six transmembrane domains are responsible for the oligomerization of LMP1, which is prerequisite for its precise function. The C-terminal regions contain two functional domains: transformation effector site 1 (TES1)/C-terminal activating region 1 (CTAR1) and TES2/CTAR2. Both domains promote cell proliferation via the NF*κ*B, AKT, and c-Jun N-terminal kinase (JNK) signaling pathways. TES1 activates NF*κ*B and AKT signaling pathways via tumor necrosis factor receptor (TNFR)-associated factors (TRAFs), whereas TES2 induces the activation of NF*κ*B and JNK/AP1 activations by stimulating the indirect assembly of TRAFs mediated by receptor-interacting protein (RIP1), TNFR-associated death domain (TRADD), and BS69. Then LMP1 is thought to be the possible cause of the increased cell proliferation in CAEBV, although there is still no direct evidence to support this.

In this study, we investigated the effect of LMP1 on an EBV-negative (Jurkat) and CAEBV-derived T (SNT16), NK (KAI3) cell lines. First, we developed two Jurkat-derived cell lines that conditionally express LMP1 and examined the effect of LMP1 on EBV-negative T cells. Then we assessed whether proliferation of T/NK cells in CAEBV requires LMP1 by inhibiting endogenous LMP1, using a dominant negative (DN) form of the protein. Although the exogenous expression of LMP1 failed to enhance proliferation in Jurkat cells, we confirmed contribution of the membrane protein to enhanced cell proliferation of T/NK cells isolated from a CAEBV patient.

## Material and Methods

### Plasmids and cell lines

LMP1 DNA derived from the B95-8 strain was subcloned into the *Hind*III and *Eco*RV sites of pTRE-Tight expression vector. The vector overexpressing DN-LMP1 was kindly provided by J. S. Pagano 11. The DN effects of this mutant LMP1 have been confirmed previously 12,13.

Jurkat is an EBV-negative T cell line derived from a human acute T-cell leukemia. The cells were cultured at 37°C with 5% CO_2_ in RPMI1640 supplemented with 10% fetal bovine serum (FBS), 4 mmol/L l-glutamine, and 100 U/mL penicillin and streptomycin. Jurkat Tet-On (JT) is a Jurkat subclone that expresses the doxycycline (Dox)-regulated transactivator, Tet-On, and was purchased from Clontech. Cell clones that conditionally express LMP1 by the addition of Dox were constructed by stable transformation of the plasmid vector described above into JT cells. Transfection was carried out by electroporation using the Invitrogen Neon transfection system following the manufacturer's recommendations. For the cloning of cell lines, the transformed cells were diluted to 0.3 cells per well and incubated in 50% conditioned medium prepared from JT cell cultures at the proliferative stage. Two JT cell lines that express LMP1 in response to Dox, JTL1-1, and JTL1-2, were successfully isolated by limited dilution. These cells were cultured with 100 *μ*g/mL G418.

SNT16 and KAI3 are an EBV-positive T and NK cell line, respectively, derived from patients of CAEBV 14,15. EBV shows latency type II in SNT16 and KAI3, and these cells expresses LMP1 endogenously 16. SNT16 and KAI3 cell lines were cultured in RPMI1640 supplemented with 10% FBS, 4 mmol/L l-glutamine, 100 U/mL penicillin and streptomycin, and 100 U/mL interleukin-2 (Primmune Inc., Kobe, Japan).

### One-step multiplex real-time RT-PCR

RNA was isolated from cells using an RNeasy Mini Kit (Qiagen, Hilden, Germany) following the manufacturer's instructions. Approximately 400 ng RNA was obtained from 5 × 10^5^ cells. LMP1 mRNA samples were quantified by one-step multiplex real-time RT-PCR using a Quantitect multiplex RT-PCR kit (Qiagen) and an Mx3000P real-time PCR system (Stratagene, La Jolla, CA) with primers and probes, as described previously 17. All samples were analyzed in triplicate. The expression of LMP1 mRNA was determined by comparing the expression of LMP1 to *β*2 microglobulin (*β*2m) mRNA as the endogenous control.

### Western blotting

Cell extracts were diluted in sample buffer (50 mmol/L Tris-HCl, pH 6.8, 2% sodium dodecyl sulfate, 10% glycerol, 6% 2-mercaptoethanol, and 0.0025% bromophenol blue) and separated by sodium dodecyl sulfate polyacrylamide gel electrophoresis (SDS-PAGE). Samples were loaded at the same protein concentration for each experiment. The primary antibodies used were anti-LMP1 antibody (S12) at 1:50, anti-actin antibody (AC-74, Sigma, St. Louis, MO) at 1:5000, anti-phospho-AKT antibody (#4058, Cell Signaling Technology, Danvers, MA) at 1:1000, anti-AKT antibody (#9272, Cell Signaling Technology) at 1:1000, anti-NF*κ*B (p65) antibody (610868, BD Biosciences, Franklin Lakes, NJ) at 1:250, anti-I*κ*B*α* antibody (#4814, Cell Signaling Technology) at 1:1000, anti-caspase-3 antibody (#9662, Cell Signaling Technology) at 1:1000, and anti-poly(ADP-ribose) polymerase (PARP) antibody (C-2-10, Sigma) at 1:2000. The secondary antibodies used were Goat Anti-Mouse Ig's HRP Conjugate (AMI3404, BioSource International, Camarillo, CA) and HRP-Goat Anti-Rabbit IgG (H+L) (656120, Invitrogen, Carlsbad, CA). The bands were visualized using WEST-oneTM Western Blot Detection System (iNtRON Biotechnology, Seongnam, Korea) or Chemi-Lumi One Super (Nacalai tesque, Kyoto, Japan).

### Cell proliferation

Cells (2 × 10^5^ per mL) were cultured for 4 days in the presence of each concentration of Dox as indicated. Live cells were counted on a hematocytometer using trypan blue exclusion at the indicated days.

### Cell cycle analysis

After the treatment with 0 or 1000 ng/mL Dox for 2 or 3 days, JT and JTL1-2 cells were fixed with 70% ethanol, and then washed with phsophate buffered saline (PBS). The fixed cells were treated with RNase, stained with 50 *μ*g/mL propidium iodide for 15 min, and then analyzed by flow cytometry using flow cytometry (FACS) Calibur (Becton Dickinson, Franklin Lakes, NJ) and ModFit LT software (Verity Software House, Topsham, ME).

### Apoptosis assay

Apoptosis was assessed by flow cytometry using a PE Annexin V Apoptosis Detection Kit I (BD Pharmingen Biosciences, Franklin Lakes, NJ) according to manufacturer's instructions. Briefly, JT and JTL1-2 cells were treated with or without 1000 ng/mL Dox for 48 h, and washed with PBS. Then they were resuspended in binding buffer, incubated in the presence of Annexin V and 7-AAD for 15 min in the dark, and then analyzed using a FACSCanto II flow cytometer and Cell Quest software (Becton Dickinson).

### Transient transfection

SNT16, Jurkat, and KAI3 cells were transfected with empty pcDNA3 vector or vector expressing DN LMP1 (DN-LMP1) under the control of CMV promoter. The DN-LMP1 has point mutations that change the PXQXT motif in the TES1 domain to AXAXT, and YYD in the tail of TES2 to IID, dysregulating its signaling activity 11. The original DNA sequence is derived from EBV B95-8 strain. The Neon transfection system (100 *μ*L Kit) (Invitrogen) was used for electroporation following the manufacturer's protocols.

## Results

### Isolation of two T-cell clones that conditionally express LMP1

**Figure 1:**
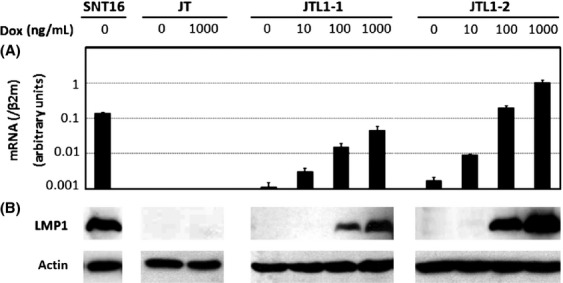
Constructions of LMP1-expressing Jurkat cells. The conditional expression of LMP1 was induced by treatment with 0, 10, 100, or 1000 ng/mL doxycycline (Dox). Cell extracts from JT, JTL1-1, and JTL1-2 cells were harvested from each experiment 2 days after the addition of Dox. (A) The quantification of LMP1 mRNA by RT-PCR. The relative expression of LMP1 mRNA is shown after normalization to *β*2 microglobulin (*β*2m) mRNA. (B) The expression of LMP1 protein was analyzed, along with actin, by western blotting.

In order to evaluate the effects of the viral oncogene, LMP1, on cell proliferation in T cells, we prepared cell clones from the Jurkat background that express LMP1 by the addition of Dox. Two clonal cell lines (JTL1-1 and JTL1-2) conditionally expressing LMP1 were obtained successfully by electroporation and limiting dilution as mentioned in the Material and Methods. The induction of LMP1 by Dox was confirmed using RT-PCR and western blotting (Fig.1). Each clone expressed LMP1 in the Dox dose-dependent manner. The LMP1 mRNA expression in JTL1-1 and JTL1-2 was comparable to the expression in CAEBV-derived cells (relative quantity to *β*2 m: SNT16, 0.14 in Fig.1A; SNT13, 0.0090 17; SNT15, 0.022 17). However, JTL1-2 induced with 1000 ng/mL Dox expressed slightly higher levels of LMP1 compared to the other CAEBV cell lines tested (Fig.1A). Therefore, JTL1-1 and JTL1-2 express an adequate range of LMP1 levels to evaluate its role in T cells.

### Expression of LMP1 failed to increase cell proliferation and growth signal intensity

**Figure 2:**
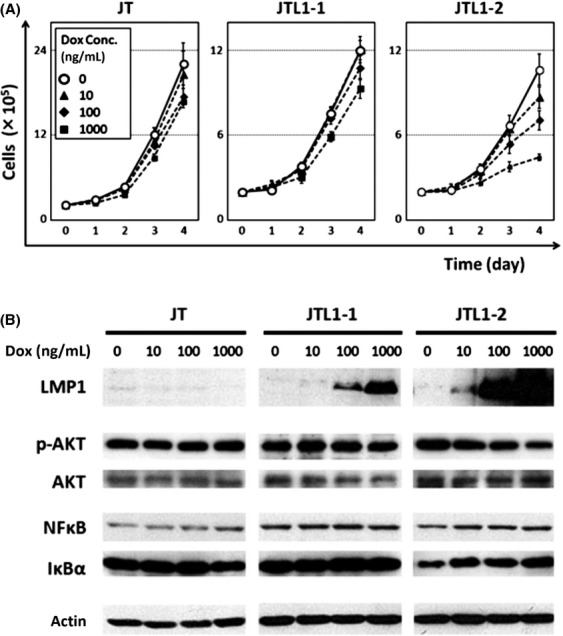
Cell proliferation and levels of signaling molecules in JT, JTL1-1, and JTL1-2 cells. (A) Cell proliferation was assessed by trypan blue staining, followed by cell counting, at days 0, 1, 2, 3, and 4 after the induction of LMP1 with 0, 10, 100, or 1000 ng/mL Dox. Experiments were performed in triplicate, and standard errors and means are shown. (B) Cell extracts harvested 2 days after Dox induction (0, 10, 100, 1000 ng/mL) were analyzed by western blotting. AKT and NF*κ*B signalings were assessed by AKT phosphorylation and the expression of NF*κ*B (p65) and I*κ*B*α*, respectively.

After successfully generating T cell lines that express LMP1 in response to Dox, the cell proliferation rates were analyzed in the presence of 0, 10, 100, or 1000 ng/mL Dox (Fig.2A). The parental cell line, JT, had the fastest growth rate of the three cell lines, with the untreated controls reaching about 20 × 10^5^ cells by day 4 (please take notice that the scale of *y*-axis is different in JT cells). The growth of JT cells slowed with increasing concentrations of Dox, suggesting that higher concentrations of Dox might be slightly toxic to the cells. Compared to JT and JTL1-1 cells, the growth of JTL1-2 cells was inhibited significantly after abundant LMP1 expression had been triggered by high concentrations of Dox (Fig.2A). This suggests that LMP1, the major oncogene of EBV, may not confer a growth advantage to T cells, at least in Jurkat cells, under conditions of exogenous expression.

We then measured the activities of AKT and NF*κ*B signaling pathways, which are activated by LMP1 in B cells. When LMP1 was expressed in a dose-dependent manner by increasing concentrations of Dox, the phosphorylation of AKT in JTL1-2 cells decreased (Fig.2B). We also assessed the levels of the p65 component of NF*κ*B and I*κ*B*α*, the major inhibitor of NF*κ*B. We found that the p65 levels were comparable, but that the expression of I*κ*B*α* increased concurrently with LMP1 expression in JTL1-2 cells (Fig.2B). The mRNA expression of I*κ*B*α*, as assessed by microarray analysis, was also upregulated in JTL1-2 cells but not in JTL1-1 cells (data not shown).

These unexpected observations reveal that LMP1 inhibits cell growth and the activation of key signaling pathways, such as AKT and NF*κ*B, in Jurkat cells, particularly when LMP1 is expressed abundantly. This contradicts previous studies that found that LMP1 induces cell proliferation through these pathways in B cells.

### LMP1-induced apoptosis in JTL1-2 cells at high concentrations of Dox

**Figure 3:**
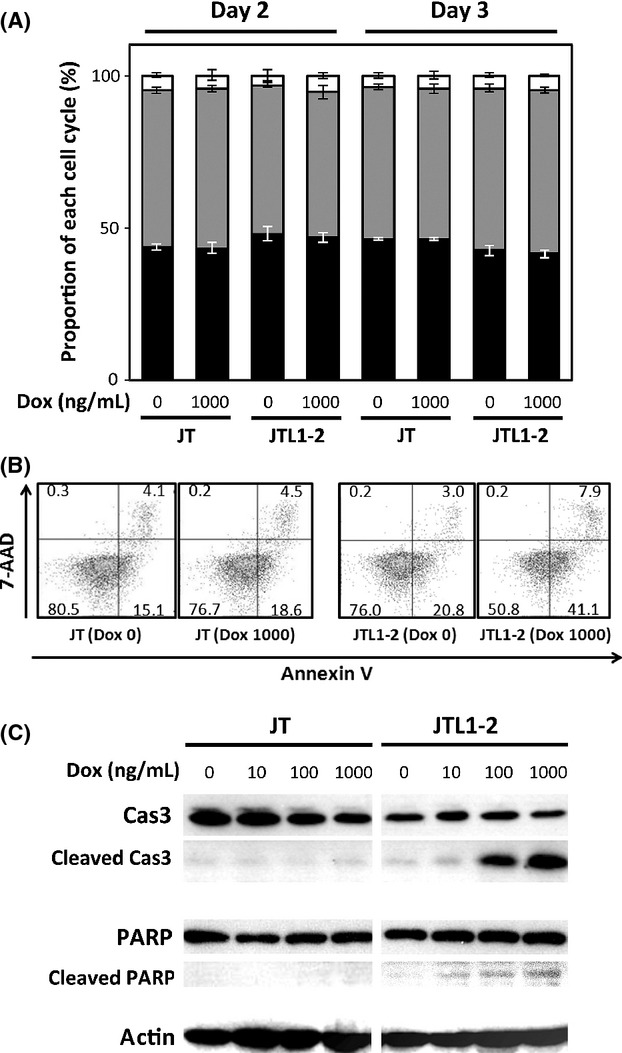
Cell cycle and apoptosis in JT and JTL1-2 cells. (A) Cell cycle analysis of JT and JTL1-2 cells was performed 2 and 3 days after induction with Dox (0 or 1000 ng/mL). Experiments were performed in triplicate and data are presented as means with standard errors. Black, gray, and white represent the ratio of cells in G1, S, and G2/M, respectively. (B) To assess the apoptosis, 2 days after the Dox induction (0 or 1000 ng/mL), JT and JTL1-2 cells were stained with 7-AAD and Annexin V and analyzed by FACS. The numbers in the corner of each quadrant indicate the percentage of cell events within the quadrant. Early apoptotic cells were defined as those positive for Annexin V but negative for 7-AAD. (C) Cell extracts harvested 2 days after Dox induction were analyzed by western blotting for the apoptosis markers, caspase-3 (Cas3) and poly(ADP-ribose) polymerase (PARP).

Because of the unexpected effects of LMP1 on the growth of JTL1-2 cells, we assessed the cause of the decreased growth rate. Therefore, cell cycle and apoptosis were examined in JTL1-2 cells in the presence or absence of Dox (Fig.3). We here did not examine cell cycle and apoptosis in JTL1-1 cells because cell growth inhibition rate of the JTL1-1 cells by Dox addition was almost comparable to the parental control cell line, JT (Fig.2A).

Propidium iodide staining followed by FACS analysis showed that the ratio of cells in G1, S, and G2/M were comparable between JT and JTL1-2 cells, with or without Dox, after 2 or 3 days of incubation (Fig.3A).

To monitor apoptotic cell death, in the Figure3B, JT or JTL1-2 cells were stained with Annexin V, an early apoptosis marker that detects the abnormal localization of phosphatidylserine on the cell membrane, and 7-AAD, which enters cells and intercalates into nuclear DNA when the integrity of cell plasma membrane has been damaged in the later stages of apoptosis. The levels of both markers were similar in JT and JTL1-2 cells without Dox treatment (Fig.3B). However, the proportion of Annexin V (+)/7-AAD (−) cells, indicative of early apoptosis execution program, increased to 41.1%, and the number of Annexin V (+)/7-AAD (+) cells, indicative of late apoptosis, also increased to 7.9% in JTL1-2 cells incubated with Dox (Fig.3B).

To confirm these observations, we carried out western blotting for caspase-3 and poly (ADP-ribose) polymerase (PARP). Caspase-3 is a cysteine protease that plays a major role in apoptosis. Caspases cleave target proteins, including PARP, during the execution of apoptosis. Western blotting indicated that the increased apoptotic cell death in JTL1-2 cells was correlated with increased cleavage of caspase-3 and PARP, whereas the total levels of these proteins were unchanged (Fig.3C). In addition, the proapoptotic gene, Jun was induced and the antiapoptotic gene, Bcl-2, were suppressed in our microarray analysis (data not shown). These results suggest that the inhibition of cell growth in JTL1-2 cells was due to the induction of apoptosis by abundant expression of LMP1.

### DN-LMP1 inhibits proliferation of CAEBV-derived cell line

**Figure 4:**
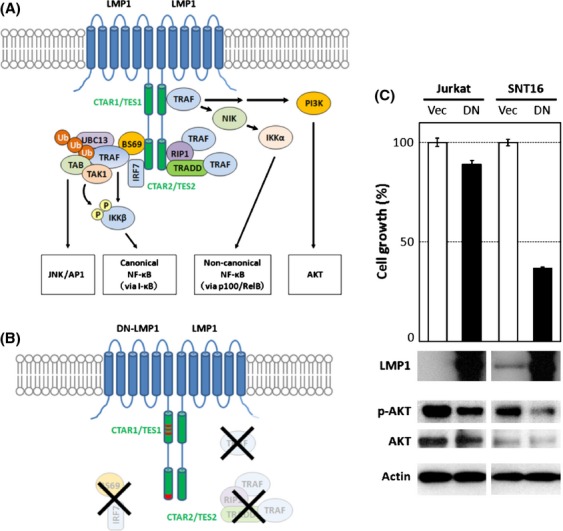
Dominant negative LMP1 inhibits proliferation of CAEBV T cells. (A) An illustration of the LMP1 signal pathway. LMP1 molecules form an oligomer that is required for its signaling activity (the oligomer is designated as a dimer for simplification). (B) DN-LMP1 has point mutations that modify the PXQXT motif in the TES1 domain to AXAXT and the YYD in the tail of TES2 to IID, resulting in the dysregulated signaling activity. (C) The growth rates of Jurkat and SNT16 cells were assessed after transient transfection with empty vector (Vec) or DN-LMP1 (DN). Experiments were performed in triplicate and data are presented as means with standard errors. Western blotting results for the expression of LMP1, phospho-AKT (pAKT), AKT, and actin are shown underneath the growth bars.

In the Jurkat T-cell background, the EBV major oncogene LMP1 did not enhance cell proliferation, and even more, it inhibited cell growth by inducing apoptotic cell death, particularly when high levels of LMP1 were produced. Because these data contradict published studies describing a proliferative role for LMP1, we used a more physiologically relevant cell line, SNT16, which is an EBV-positive cell line that was isolated from a CAEBV patient 14. The EBV in SNT16 features latency type II, and so endogenous LMP1 is produced 16. To assess whether LMP1 is necessary for T-cell proliferation in SNT16 cells, we attempted to knockdown LMP1 by siRNA and shRNA, but were unable to due to unknown technical difficulties. Therefore, we inhibited LMP1 activity using a DN form of LMP1. An LMP1 mutant with artificial point mutations in the CTAR1/TES1 and CTAR2/TES2 domains acts as a DN to inhibit the function of native LMP1, because these domains are the sites that dock to signaling mediators, such as TRAF proteins (Fig.4A and B) 11,18,19.

To test the effects of LMP1 in CAEBV, we transfected SNT16 cells with a vector expressing DN-LMP1. It is important to note that the expression of mutant LMP1 was higher than native LMP1 (Fig.4C). As a control, Jurkat cells were also transfected with DN-LMP1, in parallel. SNT16 cells transfected with DN-LMP1 grew significantly slower than empty vector controls, by approximately 40% (Fig.4C). In contrast, Jurkat cells transfected with DN-LMP1 grew only slightly slower than control (Vec), suggesting that the DN-LMP1 had little effect on the proliferation of cells lacking endogenous wild-type LMP1. The phosphorylation of AKT was correlated with the growth rate of both cell lines, suggesting that the DN-LMP1 blocked the native LMP1 signaling pathway by suppressing AKT phosphorylation (Fig.4C).

**Figure 5:**
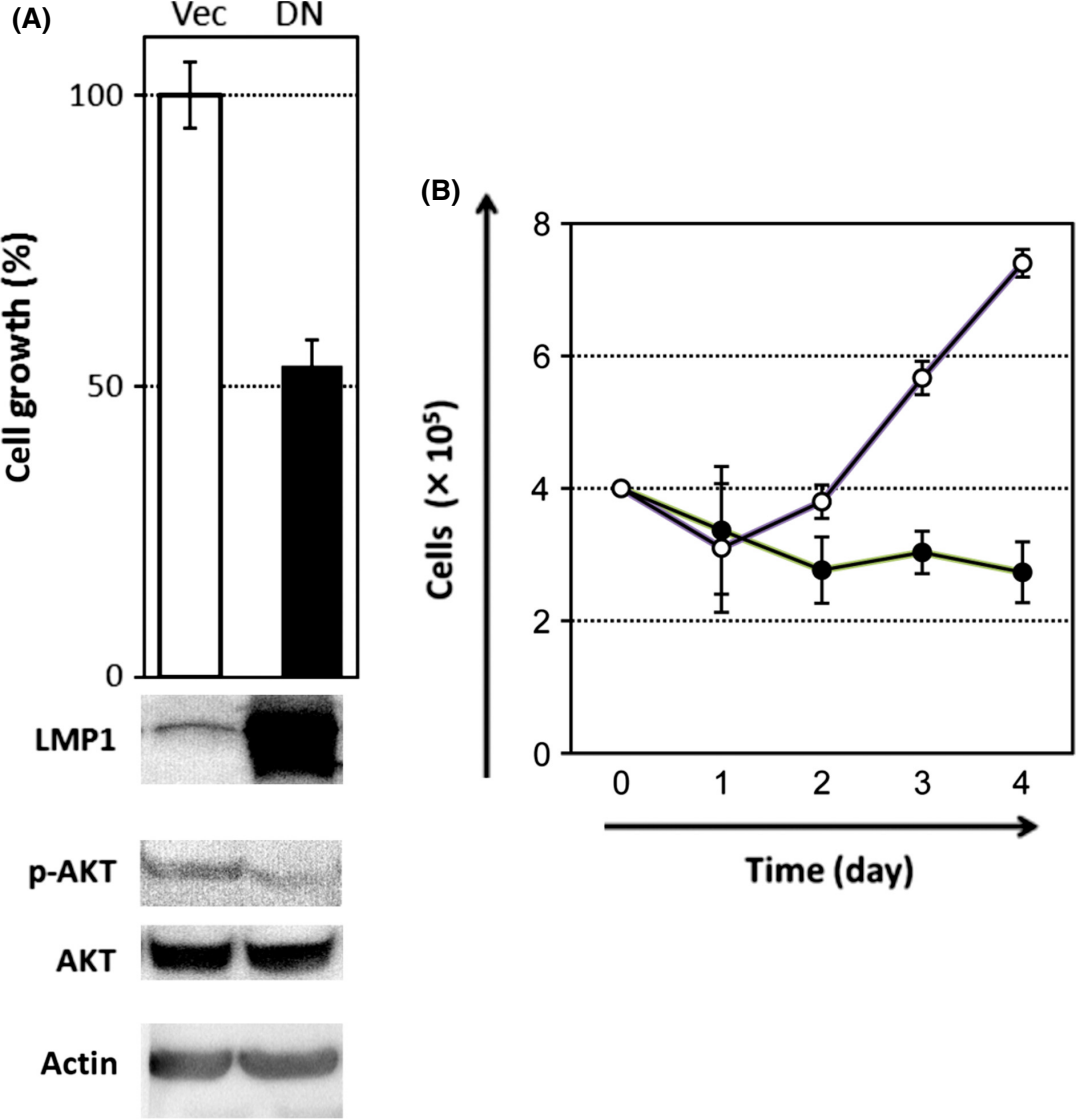
Dominant negative LMP1 inhibits proliferation of CAEBV NK cells. (A) As in Figure4, the growth rates of KAI3 cells were assessed after transient transfection with empty vector (Vec) or DN-LMP1 (DN). Experiments were performed in triplicate and data are presented as means with standard errors. Western blotting results for the expression of LMP1, phospho-AKT (pAKT), AKT, and actin are shown underneath the growth bars. (B) As in (A), KAI3 cells were transfected with empty vector (white circles) or DN-LMP1 (black circles). Cell numbers were counted on indicated days after transfection.

In order to extend these results, we then tested KAI3, an EBV-positive NK cell line derived from a CAEBV patient. Expression of DN-LMP1 caused significant decrease in growth of KAI3 cells, which correlated with weak phosphorylation of AKT (Fig.5A). When cell proliferations were monitored daily, the difference became more apparent (Fig.5B). These results suggest that LMP1 enhanced the proliferation of T/NK cells in CAEBV, similar to its effects in B cells or NPCs.

## Discussion

LMP1 is an EBV-encoded oncogene that stimulates cell growth at least in B cells and NPC. Here, we demonstrated that LMP1 regulates cell proliferation in cell lines derived from CAEBV, whereas LMP1 gave no proliferative advantage to an EBV-negative cell line.

During the preparation of this manuscript, Ndour et al. reported that a DN-LMP1 inhibited the cell growth and tumorigenesis of a T cell line artificially transformed with EBV 20,21. In contrast, we here used SNT16 and KAI3, T and NK cell lines, respectively, derived from patients with CAEBV, which are more physiologically relevant models 14. In SNT16 and KAI3 cells, EBV establishes a latent infection, expressing specific protein-coding genes including LMP1, LMP2, and EBNA1. Our results suggest that LMP1 is a necessary component of the proliferative machinery, although it is possible that LMP2 and EBNA1 also play a role. Interestingly, LMP1 and LMP2 may cooperatively promote carcinoma development in a mouse carcinogenesis model 22. The cooperation model also explains the induction of TRAF2 by LMP2 23. It is possible that LMP1 and LMP2 also act synergistically during T/NK-cell proliferation because no proliferation was stimulated in Jurkat cells expressing only LMP1 (Fig.2). Therefore, the contribution of other viral factors should be considered.

Due to unknown reasons, phosphorylated AKT in EBV-negative Jurkat cells decreased slightly by DN-LMP1 (Fig.4C). This decrease in AKT phosphorylation by DN-LMP1 might be caused by unintended influence on the AKT signaling molecules, or simply by massive expression of the protein. Anyway, the decrease in AKT phosphorylation levels in Jurkat was not potent enough to reduce the proliferation rate.

Despite a general understanding that LMP1 is an oncogene, adverse effects of LMP1 on cultured cells have also been reported in B cells, NPC cells, and other epithelial cells 24–26. It has been suggested that high levels of expression of LMP1 inhibited proliferation, and so the suppressed growth and apoptosis observed in JTL1-2 cells in our study might also be explained by the abundance of LMP1. Consistent with this, LMP1 could simultaneously induce and inhibit apoptosis in B cells, depending on the context 27. In LMP1, the C-terminal domains suppress the proapoptotic effects of transmembrane domains. Therefore, it is possible that overexpressed LMP1 in JTL1-2 cells induces apoptotic cell death by causing aggregation of the protein rather than by exerting a direct proapoptotic effect.

Expression of LMP1, either low or high levels, did not promote the proliferation of Jurkat cells, suggesting that LMP1 does not enhance the growth of these cells, regardless of the expression level. There are two possible explanations for this. One is that the intrinsic growth signals in Jurkat cell are already maximal, and so LMP1 is unable to further promote cell growth. The other is that LMP1 requires an additional factor to exert these effects. For example, LMP2 or gene products induced by the transcriptional activity of EBNA1 were not expressed in our Jurkat system.

In summary, LMP1 alone was not sufficient to enhance proliferation, at least in Jurkat cells. Therefore, LMP1 may require additional factors to promote cell growth. Nevertheless, our results suggest that LMP1 plays a central role in the lymphoproliferative disorder CAEBV. Targeting LMP1 and other factors, such as LMP2A, may facilitate effective, specific drug development for the treatment of CAEBV.
